# A meta-analysis on the uncinate fasciculus in depression

**DOI:** 10.1017/S0033291723000107

**Published:** 2023-05

**Authors:** Ellie P. Xu, Lynn Nguyen, Ellen Leibenluft, Jonathan P. Stange, Julia O. Linke

**Affiliations:** 1 Department of Psychology, University of Southern California, Los Angeles, CA, USA; 2 National Institute of Mental Health, National Institutes of Health, Bethesda, MD, USA; 3 Department of Psychiatry and Behavioral Sciences, University of Southern California, Los Angeles, CA, USA; 4 Louis A. Faillace, MD, Department of Psychiatry and Behavioral Sciences, University of Texas Health Science Center at Houston, Houston, TX, USA

**Keywords:** comorbid anxiety, depression, first-degree relatives, fractional anisotropy, radial diffusivity, uncinate fasciculus, white matter

## Abstract

Aberrant microstructure of the uncinate fasciculus (UNC), a white matter (WM) tract implicated in emotion regulation, has been hypothesized as a neurobiological mechanism of depression. However, studies testing this hypothesis have yielded inconsistent results. The present meta-analysis consolidates evidence from 44 studies comparing fractional anisotropy (FA) and radial diffusivity (RD), two metrics characterizing WM microstructure, of the UNC in individuals with depression (*n* = 5016) to healthy individuals (*n* = 18 425). We conduct meta-regressions to identify demographic and clinical characteristics that contribute to cross-study heterogeneity in UNC findings. UNC FA was reduced in individuals with depression compared to healthy individuals. UNC RD was comparable between individuals with depression and healthy individuals. Comorbid anxiety explained inter-study heterogeneity in UNC findings. Depression is associated with perturbations in UNC microstructure, specifically with respect to UNC FA and not UNC RD. The association between depression and UNC microstructure appears to be moderated by anxiety. Future work should unravel the cellular mechanisms contributing to aberrant UNC microstructure in depression; clarify the relationship between UNC microstructure, depression, and anxiety; and link UNC microstructure to psychological processes, such as emotion regulation.

Major depressive disorder (MDD), a leading cause of disability worldwide (World Health Organization, 2017), has been linked to the habitual use of dysfunctional emotion regulation strategies (Joormann & Stanton, 2016). Neurobiological models of MDD have proposed that alterations in cortical-limbic pathways contribute to these deficits in emotion regulation (Kupfer, Frank, & Phillips, 2012; Mayberg, 1997). In particular, the altered microstructure of the uncinate fasciculus (UNC), a white matter (WM) tract that connects key regions of the cortico-limbic circuit (i.e. amygdala and ventral prefrontal cortex), has been hypothesized to lead to aberrant emotion regulation in individuals with MDD (Zheng et al., 2018). However, evidence regarding perturbed UNC microstructure in individuals with MDD is mixed. Numerous studies that use a tract-of-interest (TOI) approach report atypical UNC microstructure in MDD (Table 1), but whole-brain meta-analyses yield null findings (Chen et al., 2016; Jiang et al., 2017; Liao et al., 2013; Murphy & Frodl, 2011; Zhou et al., 2022). To answer the question of whether atypical UNC microstructure represents a neurobiological mechanism and potential treatment target for depression, the present meta-analysis extends the existing literature on UNC microstructure in MDD by consolidating evidence from 44 TOI studies.

**Table 1.** Characteristics of studies comparing fractional anisotropy or radial diffusivity in the uncinate fasciculus between individuals with depression and healthy controls

Study Information			Individuals with Depression							Healthy Control Individuals		
First Author & Year	DTI Processing Pipeline	RD	*n*	Mean Age (Years)	Female (%)	Illness Duration (Years)	Taking Psychotropic Medications (%)	HDRS-17 Score	Comorbid Lifetime Anxiety (%)	*n*	Mean Age (Years)	Female (%)
**Aghajani et al. (2014)**	TBSS	X	25	15.60	84.00	–	0.00	–	72.00	21	14.70	85.71
Benedetti et al. (2011)	Tractography	X	15	50.50	66.67	19.70	100.00	–	0.00	21	46.40	47.62
**Bhatia, Henderson, Hsu, & Yim (2018)**	Tractography		103	34.50	52.43	14.10	100.00	–	–	74	39.20	48.65
**Canu et al. (2015)**	Tractography	X	71	45.04	80.28	7.75	100.00	23.25	29.58	71	45.30	80.28
**Carballedo et al. (2012)**	Tractography		37	40.40	67.57	14.10	64.86	28.20	0.00	42	36.30	59.52
Charlton et al. (2014)	Tractography	X	23	65.65	82.61	–	0.00	–	0.00	23	66.30	69.57
Choi et al. (2016)	Tractography		50	–	–	–	–	–	0.00	30	–	–
Choi et al. (2016)	Tractography		36	–	–	–	–	–	0.00	34	–	–
**Cullen et al. (2020)**	Tractography		44	15.90	25.00	–	6.80	–	45.45	37	16.30	32.40
**Davis et al. (2019)**	TBSS		165	35.73	62.78	–	0.00	–	–	103	33.20	63.90
**de Kwaasteniet et al. (2013)**	Tractography		18	44.60	77.78	–	33.33	19.17	–	24	40.20	66.67
Deng et al. (2018)	Tractography		31	30.10	58.06	3.17	0.00	25.60	–	44	28.30	38.64
**Dillon et al. (2018)**	Tractography	X	38	33.45	55.26	14.03	0.00	–	42.00	52	33.75	48.08
Doolin et al. (2019)	Tractography		14	44.21	71.43	–	85.71	24.36	–	12	40.75	66.67
**Green et al. (2021)**	TBSS		227	56.90	74.89	–	–	–	–	537	60.40	51.21
**Han et al. (2018)**	Tractography		95	43.14	80.00	3.80	53.68	15.00	–	65	40.20	69.23
**Harada et al. (2016)**	Tractography		45	60.20	57.78	10.60	91.11	–	4.44	61	62.90	72.13
Heij et al. (2019)	TBSS	X	49	48.98	34.69	–	30.61	–	42.86	39	52.10	48.72
**Hermens et al. (2018)**	Tractography		94	21.50	57.40	5.40	67.02	13.70	62.00	59	23.80	64.40
**Ho et al. (2021)**	Tractography	X	48	16.32	70.80	4.16	47.90	–	50.00	35	15.75	54.30
Jiang et al. (2015)	Tractography		35	29.54	51.43	1.07	0.00	27.70	0.00	34	31.91	50.00
**Kochunov et al. (2021)**	Tractography		2248	63.40	64.19	–	–	–	26.82	15 131	64.90	50.23
**Koreki et al. (2021)**	Tractography		15	34.60	80.00	3.40	0.00	19.70	–	27	34.70	70.37
**Korgaonkar, Williams, Song, Usherwood, & Grieve (2014)**	TBSS		80	33.80	50.00	11.30	0.00	21.00	3.75	34	31.50	47.06
**Koshiyama et al. (2020)**	TBSS	X	398	47.70	54.02	–	–	–	–	958	35.40	46.76
**Liang et al. (2019)**	Tractography		116	26.13	62.07	2.34	26.72	22.45	26.72	118	26.01	60.17
Liu et al. (2021)	Tractography		27	28.92	70.37	–	0.00	19.07	0.00	28	26.78	60.71
**Long et al. (2022)**	Tractography	X	51	31.45	100.00	–	0.00	–	0.00	49	31.86	100.00
**Mak et al. (2021)**	Tractography	X	27	23.96	66.67	2.99	0.00	–	96.30	27	22.70	62.96
**Mettenburg, Benzinger, Shimony, Snyder, & Sheline (2012)**	TBSS	X	51	68.30	68.63	–	100.00	–	–	16	68.10	68.75
Na et al. (2018)	Tractography	X	90	41.67	75.56	3.94	–	14.48	0.00	90	39.68	70.00
Ota et al. (2015)	TBSS		21	42.30	47.62	9.30	–	17.81	–	37	40.00	56.76
**Shakeel et al. (2021)**	Tractography		91	19.42	72.53	–	–	–	43.96	47	19.85	61.70
**Tatham et al. (2016)**	Tractography		55	36.40	–	11.20	0.00	21.50	0.00	18	33.20	–
Taylor et al. (2007)	Tractography		10	69.60	60.00	48.10	100.00	–	–	19	72.20	63.20
**Thomas et al. (2020)**	Tractography		17	27.76	88.24	–	–	14.88	58.82	13	24.38	61.54
**Victoria et al. (2019)**	TBSS		20	70.40	60.00	–	–	–	0.00	20	72.45	60.00
**Vilgis, Vance, Cunnington, & Silk (2017)**	Tractography	X	25	12.10	0.00	–	4.00	–	68.00	25	11.90	0.00
**Won et al. (2017)**	Tractography		103	43.08	81.55	3.71	53.40	15.08	0.00	83	39.62	68.67
Wu et al. (2020)	Tractography		36	15.60	66.67	0.84	0.00	23.19	0.00	37	15.60	51.35
**Wu et al. (2018)**	Tractography		31	28.84	58.06	1.84	0.00	23.02	0.00	62	28.69	58.06
Yuen et al. (2014)	Tractography		45	69.99	62.22	14.00	0.00	15.62	–	43	70.60	62.79
**Zhang et al. (2012) & Lamar et al. (2013)**	Tractography	X	78	55.13	60.26	–	0.00	18.76	50.00	46	57.74	52.17
**Zhang et al. (2022)**	Tractography	X	98	28.97	63.27	2.61	69.39	27.04	0.00	59	31.58	67.80
**Zheng et al. (2018)**	Tractography		20	33.50	70.00	–	0.00	22.70	–	20	34.00	70.00

**Abbreviations: DTI**, diffusion tensor imaging; **HDRS-17**, Hamilton Depression Rating Scale (17-item); **RD**, radial diffusivity.

**Note:** Bolded studies provided missing data upon request. One study (Choi et al., 2016) compared UNC FA between individuals with depression and healthy controls using two samples with different genotypes.

UNC microstructure is most commonly quantified as fractional anisotropy (FA), a metric derived from diffusion tensor imaging (DTI) that correlates positively with the directionality and coherence of fibers within a WM bundle (Basser, Mattiello, & LeBihan, 1994). Other indices used to characterize WM are axial diffusivity (AD) indicating diffusion along the main direction of the fibers, radial diffusivity (RD) measuring diffusion orthogonal to the main direction of the fibers, and mean diffusivity (MD) describing the rotationally invariant magnitude of diffusion. These three metrics have been associated with different tissue properties. AD is thought to reflect axonal organization and degeneration (Budde, Xie, Cross, & Song, 2009; Harsan et al., 2006), RD has been shown to relate to the degree of myelination in animals (Song et al., 2002), and MD has been proposed to reflect variations within the intra- and extracellular space and a reduction in neuropil (Selemon & Goldman-Rakic, 1999). Aside from FA, RD is the metric that has been most intensely studied and discussed in the context of MDD (Dillon, Gonenc, Belleau, & Pizzagalli, 2018). However, similar to the status of UNC FA, findings regarding UNC RD in depression are largely inconsistent (Table 1). Thus, it is of particular importance to integrate available evidence on these metrics (i.e., FA and RD), but integration of evidence regarding the other two metrics (i.e., AD and MD) might also advance our understanding of the neurobiological underpinnings of and potential treatment targets for depression.

Given the overarching goal of a personalized medicine approach for MDD, it is further necessary to understand which sociodemographic and clinical factors contribute to the heterogeneity in UNC findings across studies. Specifically, age might be an essential factor as the UNC matures until mid-adulthood (Lebel et al., 2012). Further, FA in the UNC has been shown to be lower in women than men (Taylor, MacFall, Gerig, & Krishnan, 2007). There might also be a diagnosis-by-sex interaction, as RD in the UNC appears to be higher in men with MDD than men without MDD (van Velzen et al., 2020).

Clinical features, such as illness duration (Jenkins et al., 2016), severity of depressive symptoms (Charlton et al., 2014; Greenberg et al., 2021), or medication use (Hu, Stavish, Leibenluft, & Linke, 2020), may also contribute to inconsistencies in UNC findings. Further, depression frequently co-occurs with anxiety. In fact, more than 50% of individuals with depression also meet the criteria for anxiety (Kessler et al., 2003). Individuals with a primary diagnosis of anxiety have also shown reduced FA in the UNC (Phan et al., 2009; Tromp et al., 2012, 2019), suggesting that atypical UNC microstructure might be a common mechanistic pathway for both depression and anxiety.

Lastly, differences in processing pipelines could contribute to variance in UNC findings (Kuchling et al., 2018). In DTI research, TOIs are defined in two ways. One strategy is to calculate the intersection between regions of a WM atlas and a sample-specific WM skeleton derived from the tract-based spatial statistics (TBSS) pipeline implemented in FSL (i.e. ENIGMA approach; http://enigma.ini.usc.edu/ongoing/dti-working-group/). A second strategy is to track fibers between relevant gray matter regions (here, amygdala and ventral prefrontal cortex; tractography approach). Notably, a recent meta-analysis using the ENIGMA approach (i.e., TBSS) did not find atypical UNC FA in individuals with MDD (van Velzen et al., 2020). However, most original studies reporting aberrant UNC microstructure in MDD use a tractography approach, which is particularly sensitive to WM alterations (Kuchling et al., 2018).

To advance our understanding of the role of atypical UNC microstructure in the risk architecture of MDD, it is further necessary to address whether such abnormalities represent a vulnerability for MDD (i.e., are also observable in individuals with an elevated risk of developing the disorder). First-degree relatives (REL) of individuals with MDD are at an increased risk for MDD themselves (Wilde et al., 2014). Indeed, earlier work suggests reduced FA in the UNC in REL compared to healthy control individuals (HC) (Huang, Fan, Williamson, & Rao, 2011). Although the number of studies in REL is limited, we believe it would be useful to synthesize this data to examine whether initial evidence supports reduced FA in the UNC as a vulnerability marker of MDD.

In summary, the present meta-analysis is the first to integrate available studies using a TOI approach to examine UNC microstructure in depression to extend existing literature in four ways. First, it consolidates evidence to determine whether reduced FA in the left and right UNC is a neurobiological marker of depression. Second, we examine whether higher RD in the left and right UNC is also a neurobiological marker of depression. Third, we test whether sociodemographic and clinical characteristics or the processing pipeline contribute to heterogeneity in UNC findings. Finally, we explore (a) whether other DTI metrics such as AD and MD also differ between individuals with and without MDD, and (b) whether atypical UNC microstructure is also present in first-degree relatives of individuals with MDD.

## Method

### Literature review

**Figure 1.**
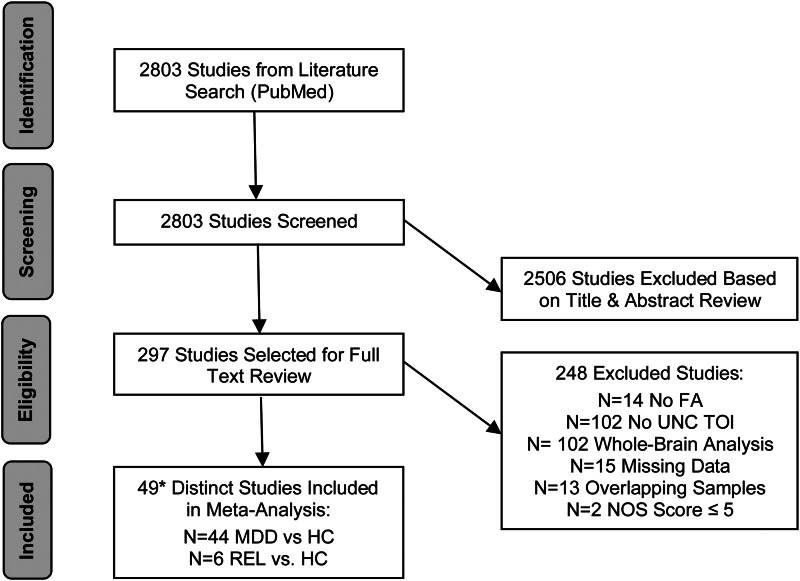
Flow chart of the literature search process. **Abbreviations: FA**, fractional anisotropy; **HC**, healthy controls; **MDD**, major depressive disorder; **NOS**, Newcastle-Ottawa Assessment Scale; **REL**, first-degree relatives; **TOI**, tract-of-interest; **UNC**, uncinate fasciculus. **Note:** *One study (Liu et al., 2021) compared UNC FA between MDD and HC as well as REL and HC.

The present meta-analysis has been pre-registered in PROSPERO (ID #CRD42021276200). To identify relevant studies, we used the search engine PubMed with the following search terms: depression and WM. We included all original research articles published before July 9th, 2022, that used a TOI approach to compare FA of the left and right UNC between individuals with a lifetime diagnosis of depression or REL and HC. Depression was broadly defined as a lifetime diagnosis of MDD, persistent depressive disorder, or other specified depressive disorders. REL were defined as first-degree relatives of an individual who either (1) met criteria for MDD, or (2) scored higher than the clinical cut-off on a dimensional depression rating scale. We excluded book chapters, conference abstracts, review articles, and case reports. We identified 49 different studies that met our inclusion criteria (Fig. 1). In addition to including individuals with current depression, one study also included individuals with lifetime depression who were currently in remission (Shakeel et al., 2021). Two studies with overlapping samples (Lamar et al., 2013; Zhang et al., 2012) were combined and included in the present meta-analysis. One study used two samples with different genotypes to compare UNC FA between MDD and HC (Choi et al., 2016); here, we included data from both samples separately.

### Data extraction

From all studies, we extracted means and standard deviations of FA and, if available, RD, MD, and AD, of the left and right UNC separately for each study group. We also pulled information about participants' mean age (43 studies, *n*_MDD_ = 4930), sex ratio (42 studies, *n*_MDD_ = 4875), mean illness duration (24 studies, *n*_MDD_ = 1339), the percentage of participants using psychotropic medication (35 studies, *n*_MDD_ = 1818), and the percentage of subjects with lifetime comorbid anxiety disorder (30 studies, *n*_MDD_ = 3803). Further, we extracted Hamilton Depression Rating Scale (HDRS; Hamilton, 1960) mean scores (23 studies, *n*_MDD_ = 1227), given that it was the most frequently reported measure of depressive symptom severity. In line with prior work, we standardized scores across different versions of the HDRS (Hu et al., 2020). Finally, we also noted whether TBSS or tractography was used to process DTI data.

We contacted corresponding authors when information regarding UNC FA or RD, or the percentage of individuals with comorbid anxiety, was missing. We obtained the requested data from 36 studies (Table 1; online Supplementary Table S1). We evaluated the quality of all studies using the Newcastle-Ottawa assessment scale (NOS; online Supplementary Table S2) and excluded two studies with NOS scores below 5 (Niida, Niida, Kuniyoshi, Motomura, & Uechi, 2013; Pines, Sacchet, Kullar, Ma, & Williams, 2018) (online Supplementary Table S3). EX initially extracted all available data, and LN cross-checked it independently.

### Data analyses

All analyses used the metafor package (version 3.4-0) for R software (R Foundation for Statistical Computing, Vienna, Austria; http://www.r-project.org/). We calculated the effect sizes for each study as standardized mean difference values (Cohen's *d*) and used the effect sizes to conduct random-effects inverse-variance weighted meta-analyses. We tested whether lower FA or higher RD in the bilateral UNC differentiated MDD or REL from HC, and considered effects significant at *p* < 0.025, correcting for tests in two hemispheres. As an exploratory analysis, we also tested whether there were differences in MD and AD in the bilateral UNC when comparing MDD and HC. Publication bias was determined using Egger's test for asymmetry, and the robustness of results was assessed using jackknife sensitivity analyses. Using a meta-analytic fixed-effects model, we compared FA effect sizes in the left and right hemispheres in individuals with MDD. Using this approach, we also indirectly compared FA effect sizes between REL and individuals with MDD to determine whether the effect size was similar in these study groups.

We conducted meta-regression analyses to investigate whether age, sex, illness duration, depressive symptom severity, medication use, comorbid anxiety, and DTI processing pipeline contributed to heterogeneity across studies. Using Bonferroni correction to correct for multiple comparisons, we set a final statistical threshold for significance of *p*_uncorrected_ < 0.007 (in other words, *p*_corrected_ < 0.05). Recommendations suggest that meta-regression analyses should include at least 20 studies to produce robust findings. Thus, we examined sources of heterogeneity for studies comparing FA between MDD to HC (*n* = 44), but not for studies comparing RD between MDD and HC (*n* = 15) or studies investigating FA between REL and HC (*n* = 6). We also did not examine sources of heterogeneity for studies comparing MD between MDD and HC (*n* = 17) or for studies comparing AD between MDD and HC (*n* = 12). Data and code supporting this study's findings are publicly available on the Open Science Forum (https://osf.io/hdp57/).

## Results

### Literature review and quality assessment

Our initial literature search encompassed 2803 studies (Fig. 1). After screening the titles and abstracts, we reviewed the full text of 297 studies. We excluded studies if they (1) did not examine FA, (2) did not include the UNC as a TOI, (3) used a whole-brain approach, (4) did not provide FA data, (5) used a sample that overlapped with another study already included in our meta-analysis, or (6) yielded a NOS quality score ≤ 5 (Fig. 1, online Supplementary Table S3). A total of 49 studies were included in our meta-analysis (Fig. 1). Overall demographic and clinical characteristics of studies included in the meta-analysis are reported in Table 2, accompanied by specific details for each study in Table 1 and online Supplementary Table S1. Meta-regression analyses were conducted in subsets of studies that provided the relevant information on age (*n* = 43), sex (*n* = 42), mean illness duration (*n* = 24), mean HDRS scores (*n* = 23), medication use (*n* = 35), and comorbid anxiety (*n* = 30). All studies provided information on the DTI processing pipeline. There were missing data on demographic characteristics (age: 1 study missing data; sex: 2 studies missing data) and clinical characteristics (mean illness duration: 20 studies missing data; mean HDRS scores: 21 studies missing data; medication use: 10 studies missing data; comorbid anxiety: 14 studies missing data).

**Table 2.** Overall demographic and clinical characteristics of studies included in the meta-analysis

	MDD *vs.* HC		REL *vs.* HC
	FA	RD	FA
Number of Studies	44	15	6
Sample Size (Total)	23 441	2619	647
Sample Size (MDD/REL)	5016	1087	116
Mean Age (Years)	38.91	38.60	19.87^b^
Female (%)	61.47	62.00	51.02^a^
Illness Duration (Years)	8.89^a^	7.88^a^	0.00^a^
Medicated (%)	32.42	34.76	–
Mean HDRS-17 Score	20.58^a^	20.88^b^	3.23^b^
Comorbid Anxiety (%)	23.31^a^	34.67	–

**Abbreviations**: **FA**, fractional anisotropy; **HC**, healthy controls; **HDRS-17**, Hamilton Depression Rating Scale (17-item); **MDD**, major depressive disorder; **RD**, radial diffusivity; **REL**, first-degree relatives at-risk for depression.

a These means are based on less than 75% of studies, which reported this information in the published manuscript.

b These means are based on less than 50% of studies, which reported this information in the published manuscript.

### Fractional anisotropy in the uncinate fasciculus

**Figure 2.**
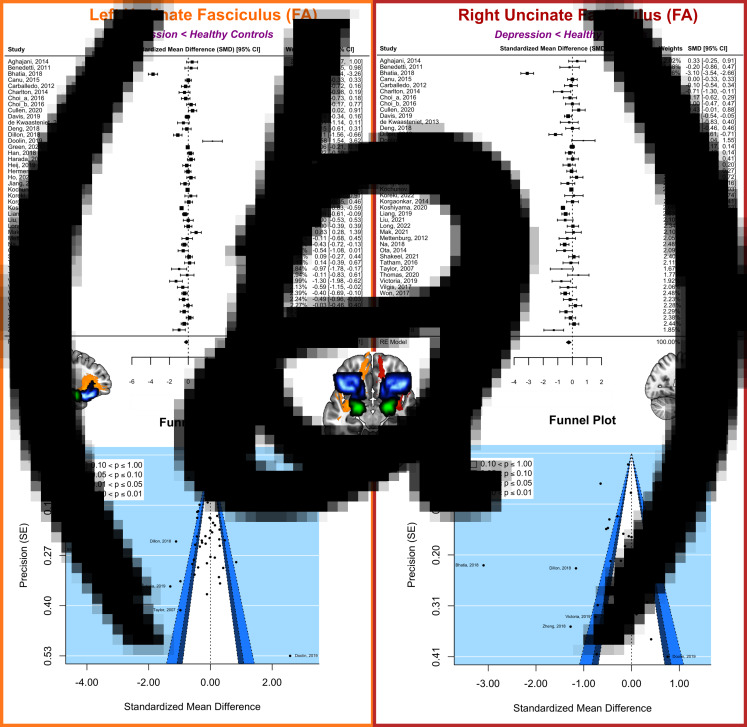
Tract-of-interest meta-analysis comparing fractional anisotropy in the uncinate fasciculus between individuals with depression and healthy controls. Here, the left uncinate fasciculus (in orange) and right uncinate fasciculus (in red) are depicted. These tracts interconnect the amygdala (in green) to the orbitofrontal cortex (in blue). (*a*) Forest plots, with the black diamond representing the overall effect size of each study. (*b*) Funnel plots, with the dotted line representing the overall effect size. Symmetric funnel plots indicate an absence of publication bias, with a majority of studies falling in the area of statistical non-significance (*p* > 0.1). The five most extreme points on each funnel plot are labeled with the study author and year. **Abbreviations: CI**, confidence interval; **FA**, fractional anisotropy; **RE model**, random-effect model; **SE**, standard error.

The Egger's test indicated no publication bias among the studies that reported FA in the left (*z* = 0.79, *p* = 0.432; Figure 2) and right UNC (*z* = −0.01, *p* = 0.993; Figure 2), so we included all studies in the final analysis. We found that individuals with MDD showed reduced FA in the right UNC (WMD = −0.25, 95% CI [−0.42 to −0.09], *p* = 0.003; Figure 2); however, this effect was only marginally significant in the left UNC (WMD = −0.21, 95% CI [−0.42 to 0.01], *p* = 0.059; Figure 2). FA effect sizes between the left and right UNC did not differ (*p* = 0.735), suggesting no significant difference between the two hemispheres. In the right UNC, jackknife sensitivity analysis found that no single study drove these results. For the left UNC, jackknife sensitivity analysis showed that the effect became significant (*p* = 0.013) when leaving out one particular study, which consisted of individuals with MDD who experienced moderate anxiety symptoms (Doolin et al., 2019).

### Heterogeneity in fractional anisotropy findings

Comorbid anxiety explained variability in findings in the left (*β* = 0.01, *z* = 2.00, *R*^2^ = 15%, *p*_uncorrected_ = 0.046, *p*_corrected_ = 0.322) but not right UNC (*β* = 0.00, *z* = 1.46, *R*^2^ = 4%, *p*_uncorrected_ = 0.146, *p*_corrected_ ≥ 1; online Supplementary Table S4). Specifically, comorbid anxiety attenuated the group difference between individuals with MDD and HC in the left UNC. We found no evidence that age, sex, duration of illness, the severity of depressive symptoms, medication use, or the DTI processing pipeline contributed to variance in UNC findings in individuals with MDD (all *ps*_uncorrected_ > 0.124, all *ps*_corrected_ > 0.868).

### Radial diffusivity in the uncinate fasciculus

For the left UNC, Egger's test indicated one outlier that showed greatly elevated RD in individuals with MDD (Koshiyama et al., 2020). After excluding this study, Egger's test showed no publication bias in the left UNC (*z* = −0.73, *p* = 0.466; online Supplementary Fig. S1). In the right UNC, Egger's test suggested no publication bias (*z* = −0.72, *p* = 0.475; online Supplementary Fig. S1). Thus, for the final analysis, we excluded one study (Koshiyama et al., 2020) for the left UNC and included all studies for the right UNC. We found no differences in RD in the left (WMD = −0.01, 95% CI [−0.19 to 0.17], *p* = 0.936; online Supplementary Fig. S1) or right UNC (WMD = 0.11, 95% CI [−0.10 to 0.31], *p* = 0.307; online Supplementary Fig. S1) when comparing individuals with MDD to HC. RD effect sizes were comparable between the left and right UNC (*p* = 0.416).

### Exploratory analysis

We found no FA differences in the left or right UNC when comparing REL to HC (online Supplementary Fig. S2). Indirect comparisons of UNC FA between hemispheres also yielded no significant results (*p* = 0.811). However, indirect comparisons of UNC FA between REL and individuals with MDD yielded significant results in the right UNC (WMD = 0.33, 95% CI [0.06 to 0.60], *p* = 0.019) and marginally significant results in the left UNC (WMD = 0.33, 95% CI [−0.05 to 0.71], *p* = 0.093). In other words, individuals with MDD showed reduced UNC FA compared to REL. Separately, we found no differences in MD or AD in the left or right UNC when comparing individuals with MDD to HC (online Supplementary Figs S3 and S4).

## Discussion

The present meta-analysis, which integrates data from 5016 individuals with MDD and 18 425 HC, associates MDD with reduced FA in the uncinate fasciculus, suggesting perturbed coherence of this tract. We also provide preliminary evidence for a laterality effect potentially related to comorbid anxiety. In contrast to our hypothesis, this effect was not related to alterations in RD, which was comparable between individuals with (*N*_MDD_ = 1087) and without MDD (*N*_HC_ = 1532). We also found no support for the hypothesis that aberrant UNC FA represents a vulnerability for MDD. However, the available sample of REL was small (*N*_REL_ = 116, *N*_HC_ = 531), and thus must be cautiously interpreted.

Consistent with neurobiological models of depression (Kupfer et al., 2012; Mayberg, 1997) that state that aberrancies in cortical-limbic pathways are implicated in perturbed emotion regulation, we associate MDD with reduced FA in the UNC, which links the ventral prefrontal cortex and the amygdala. Prior work links reduced FA in the UNC to less effective reappraisal (d'Arbeloff et al., 2018; Eden et al., 2015; Zuurbier, Nikolova, Åhs, & Hariri, 2013) and less amygdala regulation in response to emotional stimuli (Hein et al., 2018). However, these studies focused on non-depressed individuals. Thus, future studies should investigate the relationship between UNC microstructure and emotion regulation deficits in individuals with MDD, possibly by integrating DTI and task-based functional magnetic resonance imaging.

Integrating information from 44 TOI studies, we extend findings from a prior meta-analysis that found reduced UNC FA when examining three whole-brain and TOI studies on individuals with late-life depression (Wen, Steffens, Chen, & Zainal, 2014). Specifically, we provide more conclusive evidence of reduced UNC FA in individuals with depression across the lifespan. However, our meta-analysis contrasts prior whole-brain meta-analyses that found no alterations in UNC FA in individuals with MDD (Chen et al., 2016; Jiang et al., 2017; Liao et al., 2013; Murphy & Frodl, 2011; Zhou et al., 2022). Indeed, a recent report (Winter et al., 2022) shows a large overlap in the distribution of neurobiological markers including whole-brain FA between individuals with depression and HCs. However, unlike TOI approaches, whole-brain approaches are less sensitive to effects in small tracts such as the UNC. In contrast to a recent meta-analysis that also found no differences in UNC FA between individuals with MDD and HC using the ENIGMA approach (van Velzen et al., 2020), 80% of the studies included in our meta-analysis used a tractography (rather than a TBSS) approach, which has been shown to be more sensitive to capturing WM alterations (Kuchling et al., 2018). Thus, methodological heterogeneity might explain diverging findings in meta-analyses of WM in MDD.

Our findings link MDD most strongly to reduced FA in the right UNC. Effects were less consistent in the left hemisphere. Our findings suggest that comorbid anxiety might contribute to this heterogeneity. Specifically, a higher proportion of patients with comorbid anxiety was associated with higher FA in the left UNC. This finding is consistent with prior work demonstrating a positive relationship between trait anxiety and FA in the left UNC (Modi et al., 2013; Montag, Reuter, Weber, Markett, & Schoene-Bake, 2012). This finding is also consistent with a prior study, which found that individuals with MDD and comorbid anxiety do not show altered UNC microstructure (Canu et al., 2015), though individuals with solely MDD (Table 1) or anxiety (Tromp et al., 2012) do show alterations in the UNC. More work is needed to delineate specific and shared mechanisms of depression and anxiety. Such work might leverage latent variable approaches such as bifactor models (Scopel Hoffmann et al., 2022), which can be used to parse specific and shared factors of symptoms of depression and anxiety, which can then be related to WM microstructure.

Perturbations in UNC microstructure are not specific to MDD. For example, atypical UNC microstructure has been linked to bipolar disorder (Xu et al., 2022), anxiety disorders (Jenkins et al., 2016), and attention-deficit/ hyperactivity disorder (van Ewijk, Heslenfeld, Zwiers, Buitelaar, & Oosterlaan, 2012). A mega-analysis also found atypical UNC microstructure in individuals with schizophrenia (Koshiyama et al., 2020). As the UNC is implicated in the pathophysiology of different disorders, it is pivotal that future work examines more closely its relevance to specific transdiagnostic processes (e.g. emotion regulation).

FA is a sensitive but non-specific indicator of WM microstructure. At a cellular level, FA findings might relate to atypical myelination. Indeed, it has been proposed that myelination, better assessed through RD, is a mechanism of depression (Boda, 2021; Gao et al., 2017; Hemanth Kumar et al., 2014; Hou et al., 2021). Our results do not support this hypothesis. Thus, future work must investigate alternative drivers of aberrant FA, such as vascular and glial changes and axonal branching or pruning (Sampaio-Baptista & Johansen-Berg, 2017). Identifying cellular mechanisms underlying perturbed UNC microstructure in MDD might aid in developing novel therapeutics for this debilitating condition.

Finally, it is essential to place atypical UNC microstructure within the risk architecture of MDD. To this end, we integrated studies examining UNC microstructure in REL of MDD. Differences in UNC microstructure were observed between REL and individuals with MDD, but not between REL and HC. Though preliminary, this suggests that altered UNC microstructure may not represent a vulnerability but develops during MDD, and thus may be a consequence, or a ‘scar’, of MDD (Rohde, Lewinsohn, & Seeley, 1990; Wichers, Geschwind, van Os, & Peeters, 2010).

Numerous studies have shown that repetition of behaviors and sleep patterns independently modify WM throughout the lifespan (Sampaio-Baptista & Johansen-Berg, 2017). It is conceivable that, during MDD, the well-documented bias towards negative emotional content (Gotlib & Joormann, 2010) and the excessive use of maladaptive emotion regulation strategies such as rumination (Joormann & Stanton, 2016) shape the UNC. Further, perturbed sleep is a frequently reported symptom of depression (Nutt, Wilson, & Paterson, 2008), that often persists during remission. Sleep deprivation has been hypothesized to lead to reduced structural integrity of the UNC (Jamieson, Broadhouse, Lagopoulos, & Hermens, 2020), which is supported by recent findings that associated poor sleep quality with reduced FA and higher RD in the UNC in healthy adolescents (Jamieson et al., 2021). Future work should investigate the relationship between UNC FA and sleep in individuals with MDD, as understanding this association could have implications for the treatment of MDD.

Finally, we like to point out that depression as a diagnostic category might encompass multiple subtypes (Fried, 2017). Thus, it may not be possible to link such a broad phenotype to a specific neurobiological signature (Winter et al., 2022). Instead, future work might consider, alternate phenotyping strategies such as latent profile analysis, when examining the role of WM microstructure in depression, as perturbations in the UNC might only be found in subgroups of patients. This could be an important step towards a personalized treatment approach for depression.

### Limitations

While we were well-powered to examine our main question, findings on RD, MD, AD and REL must be considered preliminary, given the substantially smaller number of studies used in these analyses. Unfortunately, we could not determine whether findings generalize to diverse racial and ethnic backgrounds as the majority of studies (*n* = 40) did not report these variables. It will be important that future studies report participants' racial and ethnic background, as these demographic features may relate to traumatic experiences (Kirkinis, Pieterse, Martin, Agiliga, & Brownell, 2021) and could contribute to inter-study variance in UNC findings.

## Conclusion

The present meta-analysis found reduced FA in the UNC in individuals with, but not those at risk for, MDD. However, comorbid anxiety may weaken associations between depression and UNC microstructure. Future work needs to investigate when and how perturbations in UNC microstructure develop and should aim to link it to psychological processes previously implicated in depression, such as impaired emotion regulation.

## Supplementary material

For supplementary material accompanying this paper visit https://doi.org/10.1017/S0033291723000107. click here to view supplementary material
