# Supplementary material for: A meta-analysis on the uncinate fasciculus in depression
Source: Psychol Med. 2023 Apr 13;53(7):2721–31. doi: 10.1017/S0033291723000107 (PMC10235669; doi:10.1017/S0033291723000107)
Supplement: Supplementary file 1 [file S0033291723000107sup001.docx]

**A meta-analysis on the uncinate fasciculus in depression**

***Supplemental Information***

One study included individuals with current depression and individuals with lifetime depression, who were currently in remission.^1^ When this study was included in analyses, we found that individuals with MDD showed reduced FA in the right UNC (WMD = −0.25, 95% CI = [−0.42, −0.09], p = 0.003) and marginally reduced FA in the left UNC (WMD = −0.21, 95% CI = [−0.42, 0.01], p = 0.059). When the study was excluded, we also found that individuals with MDD showed reduced FA in the right UNC (WMD = −0.25, 95% CI = [−0.42, −0.09], p = 0.003) and marginally reduced FA in the left UNC (WMD = −0.21, 95% CI = [−0.43, 0.01], p = 0.056).

| Table S1. Characteristics of studies comparing fractional anisotropy in the uncinate fasciculus between first-degree relatives at-risk for depression and healthy control individuals. | | | | | | | | |
| --- | --- | --- | --- | --- | --- | --- | --- | --- |
|  | **First-Degree Relatives**  **Healthy Control Individuals** | | | | | | | |
| Study | ***n*** | **Index Relationship** | **Mean Age (Years)** | **Female (%)** | **Illness Duration (Years)** | ***n*** | **Mean Age (Years)** | **Female (%)** |
| Borchers, 2021 ^2^ | 6 | Offspring | - | - | 0 | 32 | - | - |
| Hay, 2020 ^3^ | 5 | Offspring | - | - | 0 | 49 | - | - |
| Hung, 2017 ^4^ | 20 | Offspring | 11.10 | 50.00 | 0 | 20 | 10.65 | 50.00 |
| Lautarescu, 2022 ^5^ | 52 | Offspring | - | - | 0 | 361 | - | - |
| Liu, 2021 ^6^ | 16 | Unspecified | 30.93 | 68.75 | 0 | 28 | 26.78 | 60.71 |
| Versace, 2018 ^7^ | 17 | Offspring | - | - | - | 41 | - | - |

**^Note:^** ^Bolded studies provided missing data upon request. Studies missing information on age and sex reported these variables for the total sample, but not for the individual study groups (first-degree relatives and healthy controls) separately.^

| **Table S2.** Newcastle-Ottawa Quality Assessment Scale (Adapted for this Meta-Analysis). A study can be awarded a maximum of one point for each numbered item within the Selection and Exposure categories. A maximum of two points can be given for Comparability. | | | |
| --- | --- | --- | --- |
| **Selection** | | 1. Is the case definition (diagnosis of depression) adequate? 2. **1 point: clinician interview was conducted** (e.g., SCID, K-SADs, DIGS, NIMH DIS) 3. **0.5 points: clinician interview was conducted by an undergraduate student,** or diagnosis was assessed by a psychiatrist in accordance with DSM or ICD criteria 4. **0 points**: chart review or self-report or MINI   Is the case definition (relative of individual with depression) adequate?   1. **1 point: clinician interview was conducted** (e.g., SCID, K-SADs, MINI) 2. **0.5 points: clinical interview was conducted by an undergraduate student** 3. **0 points:** chart review or self-report 4. Representativeness of cases (diagnosis of depression) 5. **1 point: broad inclusion criteria,** e.g., *all* youth or *all* adults with MDD, or patients with MDD identified from inpatient and outpatient visits 6. **0 points**: restrictive exclusion criteria, e.g., only age 60+ adults with MDD, only unmedicated patients with MDD, only patients with MDD taking antidepressants, or only patients with MDD with no other psychiatric comorbidities   Representativeness of cases (relative of individual with depression)   1. **1 point: broad inclusion criteria,** e.g., *all* first-degree relatives 2. **0 points:** restrictive exclusion criteria, e.g., only siblings; only offspring 3. Selection of healthy controls 4. **1 point: healthy controls from the community** 5. **0 points:** healthy controls from the hospital; no description about healthy controls 6. Definition of healthy controls 7. **1 point: no history of depression or other psychiatric disorders** 8. **0 points:** no description | |
| **Comparability** | | 1. Comparability of cases and healthy controls on basis of design or analysis    1. **2 points: study controls for two confounding variables (e.g., age and sex)** either by matching during patient and control recruitment, by adjusting for them in the analysis, or by including them as dependent variables in the analysis **OR** study states that there is no significant difference between the groups for confounders (i.e., age and sex)    2. **1 point: no significant difference between groups for only one confounding variable** (e.g., age) but not a second confounding variable (e.g., sex) **OR** only one confounding variable (e.g., age) is used as a covariate in the analysis    3. **0 points**: no mention of whether there is a significant difference in two confounding variables (e.g., age and sex) between groups, and the confounding variables are not included in statistical analyses | |
| **Exposure** | | 1. Ascertainment of exposure    1. **1 point**: DTI data acquired; data processed and analyzed in blinded fashion   **Note: ALL studies received 1 point for this criterion**   1. Same method of ascertainment (i.e., DTI imaging) for cases and healthy controls    1. **1 point**: **ALL studies received 1 point for this criterion** 2. Non-response rate    1. **1 point: no patient DTI data was excluded due to motion artifact or other reasons** **OR** an equal proportion of data was excluded for each study group    2. **0 points**: inverse of the above statement | |
| \| **Study Information** \| \| \| **Quality Indications** \| \| \| \| \| \| \| \| \| \| \| --- \| --- \| --- \| --- \| --- \| --- \| --- \| --- \| --- \| --- \| --- \| --- \| --- \| \| **First Author** \| **Year** \| **Group** \| **A** \| **B** \| **C** \| **D** \| **E** \| **F** \| **G** \| **H** \| **Total** \| \| Aghajani \| 2014 \| MDD \| 0.5 \| 0 \| 1 \| 1 \| 2 \| 1 \| 1 \| 1 \| 7.5 \| \| Benedetti \| 2011 \| MDD \| 1 \| 0 \| 0 \| 1 \| 2 \| 1 \| 1 \| 1 \| 7 \| \| Bhatia \| 2018 \| MDD \| 0.5 \| 0 \| 1 \| 0 \| 2 \| 1 \| 1 \| 0 \| 5.5 \| \| Canu \| 2015 \| MDD \| 1 \| 0 \| 1 \| 1 \| 2 \| 1 \| 1 \| 1 \| 8 \| \| Carballedo \| 2012 \| MDD \| 1 \| 0 \| 1 \| 1 \| 2 \| 1 \| 1 \| 1 \| 8 \| \| Charlton \| 2014 \| MDD \| 1 \| 0 \| 1 \| 1 \| 2 \| 1 \| 1 \| 0 \| 7 \| \| Choi \| 2016 \| MDD \| 1 \| 0 \| 1 \| 1 \| 2 \| 1 \| 1 \| 1 \| 8 \| \| Cullen \| 2020 \| MDD \| 1 \| 0 \| 1 \| 1 \| 2 \| 1 \| 1 \| 0 \| 7 \| \| Davis \| 2019 \| MDD \| 0 \| 0 \| 1 \| 1 \| 2 \| 1 \| 1 \| 0 \| 6 \| \| de Kwaasteniet \| 2013 \| MDD \| 0.5 \| 0 \| 1 \| 1 \| 2 \| 1 \| 1 \| 1 \| 7.5 \| \| Deng \| 2018 \| MDD \| 1 \| 0 \| 0 \| 1 \| 2 \| 1 \| 1 \| 0 \| 6 \| \| Dillon \| 2018 \| MDD \| 1 \| 0 \| 0 \| 1 \| 2 \| 1 \| 1 \| 0 \| 6 \| \| Doolin \| 2019 \| MDD \| 0.5 \| 0 \| 1 \| 0 \| 2 \| 1 \| 1 \| 1 \| 6.5 \| \| Green \| 2021 \| MDD \| 1 \| 1 \| 1 \| 1 \| 2 \| 1 \| 1 \| 1 \| 9 \| \| Han \| 2018 \| MDD \| 1 \| 0 \| 1 \| 1 \| 2 \| 1 \| 1 \| 1 \| 8 \| \| Harada \| 2016 \| MDD \| 0 \| 0 \| 1 \| 0 \| 2 \| 1 \| 1 \| 1 \| 6 \| \| Heij \| 2019 \| MDD \| 0.5 \| 0 \| 1 \| 1 \| 2 \| 1 \| 1 \| 0 \| 6.5 \| \| Hermens \| 2018 \| MDD \| 1 \| 0 \| 1 \| 1 \| 2 \| 1 \| 1 \| 1 \| 8 \| \| Ho \| 2021 \| MDD \| 1 \| 0 \| 1 \| 1 \| 2 \| 1 \| 1 \| 1 \| 8 \| \| Jiang \| 2015 \| MDD \| 1 \| 0 \| 1 \| 1 \| 2 \| 1 \| 1 \| 1 \| 8 \| \| Kochunov \| 2021 \| MDD \| 0 \| 1 \| 1 \| 0 \| 2 \| 1 \| 1 \| 0 \| 6 \| \| Koreki \| 2022 \| MDD \| 1 \| 0 \| 0 \| 1 \| 2 \| 1 \| 1 \| 1 \| 7 \| \| Korgaonkar \| 2014 \| MDD \| 0 \| 0 \| 1 \| 0 \| 2 \| 1 \| 1 \| 1 \| 6 \| \| Koshiyama \| 2020 \| MDD \| 1 \| 0 \| 1 \| 1 \| 2 \| 1 \| 1 \| 0 \| 7 \| \| Lamar \| 2013 \| MDD \| 0.5 \| 0 \| 1 \| 0 \| 2 \| 1 \| 1 \| 0 \| 5.5 \| \| Liang \| 2019 \| MDD \| 1 \| 0 \| 0 \| 0 \| 2 \| 1 \| 1 \| 1 \| 6 \| \| Liu \| 2021 \| MDD \| 1 \| 0 \| 0 \| 0 \| 2 \| 1 \| 1 \| 1 \| 6 \| \| Long \| 2022 \| MDD \| 0.5 \| 0 \| 0 \| 1 \| 2 \| 1 \| 1 \| 1 \| 6.5 \| \| Mak \| 2021 \| MDD \| 1 \| 0 \| 1 \| 1 \| 2 \| 1 \| 1 \| 1 \| 8 \| \| Mettenburg \| 2012 \| MDD \| 1 \| 0 \| 0 \| 1 \| 2 \| 1 \| 1 \| 1 \| 7 \| \| Na \| 2018 \| MDD \| 1 \| 0 \| 0 \| 1 \| 2 \| 1 \| 1 \| 1 \| 7 \| \| Niida \| 2013 \| MDD \| 0.5 \| 0 \| 0 \| 0 \| 1 \| 1 \| 1 \| 1 \| 4.5 \| \| Ota \| 2015 \| MDD \| 0.5 \| 1 \| 1 \| 1 \| 2 \| 1 \| 1 \| 1 \| 8.5 \| \| Pines \| 2018 \| MDD \| 0 \| 0 \| 1 \| 0 \| 1 \| 1 \| 1 \| 0 \| 4 \| \| Shakeel \| 2021 \| MDD \| 0.5 \| 0 \| 1 \| 1 \| 2 \| 1 \| 1 \| 0 \| 6.5 \| \| Tatham \| 2016 \| MDD \| 1 \| 0 \| 1 \| 0 \| 2 \| 1 \| 1 \| 1 \| 7 \| \| Taylor \| 2007 \| MDD \| 1 \| 0 \| 1 \| 0 \| 2 \| 1 \| 1 \| 1 \| 7 \| \| Thomas \| 2020 \| MDD \| 0.5 \| 0 \| 1 \| 0 \| 2 \| 1 \| 1 \| 1 \| 6.5 \| \| Victoria \| 2019 \| MDD \| 1 \| 0 \| 1 \| 1 \| 2 \| 1 \| 1 \| 1 \| 8 \| \| Vilgis \| 2017 \| MDD \| 1 \| 0 \| 1 \| 0 \| 2 \| 1 \| 1 \| 1 \| 7 \| \| Won \| 2017 \| MDD \| 1 \| 0 \| 0 \| 1 \| 2 \| 1 \| 1 \| 1 \| 7 \| \| Wu \| 2020 \| MDD \| 1 \| 0 \| 0 \| 1 \| 2 \| 1 \| 1 \| 1 \| 7 \| \| Wu \| 2018 \| MDD \| 1 \| 0 \| 0 \| 1 \| 2 \| 1 \| 1 \| 1 \| 7 \| \| Yuen \| 2014 \| MDD \| 0 \| 0 \| 1 \| 1 \| 2 \| 1 \| 1 \| 1 \| 7 \| \| Zhang \| 2012 \| MDD \| 0.5 \| 0 \| 1 \| 0 \| 2 \| 1 \| 1 \| 1 \| 6.5 \| \| Zhang \| 2022 \| MDD \| 1 \| 0 \| 1 \| 1 \| 2 \| 1 \| 1 \| 0 \| 7 \| \| Zheng \| 2018 \| MDD \| 1 \| 0 \| 0 \| 1 \| 2 \| 1 \| 1 \| 1 \| 7 \| \| Borchers \| 2021 \| REL \| 0 \| 0 \| 1 \| 1 \| 2 \| 1 \| 1 \| 1 \| 7 \| \| Hay \| 2020 \| REL \| 0 \| 0 \| 1 \| 1 \| 2 \| 1 \| 1 \| 1 \| 7 \| \| Hung \| 2017 \| REL \| 1 \| 0 \| 1 \| 1 \| 2 \| 1 \| 1 \| 0 \| 7 \| \| Lautarescu \| 2022 \| REL \| 0 \| 0 \| 1 \| 1 \| 2 \| 1 \| 1 \| 1 \| 7 \| \| Liu \| 2021 \| REL \| 1 \| 1 \| 0 \| 0 \| 2 \| 1 \| 1 \| 1 \| 7 \| \| Versace \| 2018 \| REL \| 1 \| 0 \| 1 \| 0 \| 2 \| 1 \| 1 \| 0 \| 6 \|   **Table S3.** Quality assessment using the Newcastle-Ottawa Scale.  **^Abbreviations: A.^** ^Adequate case definition;^ **^B.^** ^Representativeness of cases;^ **^C.^** ^Selection of healthy controls;^ **^D.^** ^Definition of healthy controls;^ **^E.^** ^Comparability of patients and healthy controls based on study design or analysis;^ **^F.^** ^Ascertainment of exposure;^ **^G.^** ^Same method of ascertainment for cases and healthy controls;^ **^H.^** ^Comparable proportion of diffusion tensor imaging data excluded in cases versus healthy controls.^ | |  |  |

**Table S4.** Coefficient estimates and uncorrected p-values for null findings on demographic and clinical characteristics as sources of heterogeneity in UNC findings.

|  | **Left UNC**  **β p** | | **Right UNC**  **β p** | | |
| --- | --- | --- | --- | --- | --- |
| **Age** | -0.01 | 0.484 | | -0.01 | 0.206 |
| **Sex Ratio (% Women)** | 0.01 | 0.218 | | 0.00 | 0.368 |
| **Illness Duration** | -0.03 | 0.167 | | -0.02 | 0.124 |
| **Depressive Symptom Severity** | -0.00 | 0.970 | | 0.02 | 0.136 |
| **Medication Use** | 0.00 | 0.493 | | 0.00 | 0.351 |
| **Comorbid Lifetime Anxiety** | 0.01 | 0.046 | | 0.00 | 0.146 |
| **DTI Processing Pipeline (TBSS)** | -0.08 | 0.770 | | -0.03 | 0.899 |
| **^Abbreviations:^** **^DTI^**^, diffusion tensor imaging;^ **^TBSS,^** ^tract-based spatial statistics;^ **^UNC^**^, uncinate fasciculus.^ | | | | | |

**Figure S1. Tract-of-interest meta-analysis comparing radial diffusivity in the uncinate fasciculus between individuals with depression and healthy controls. Here, the left uncinate fasciculus (in orange) and right uncinate fasciculus (in red) are depicted. These tracts interconnect the amygdala (in green) to the orbitofrontal cortex (in blue).** ^
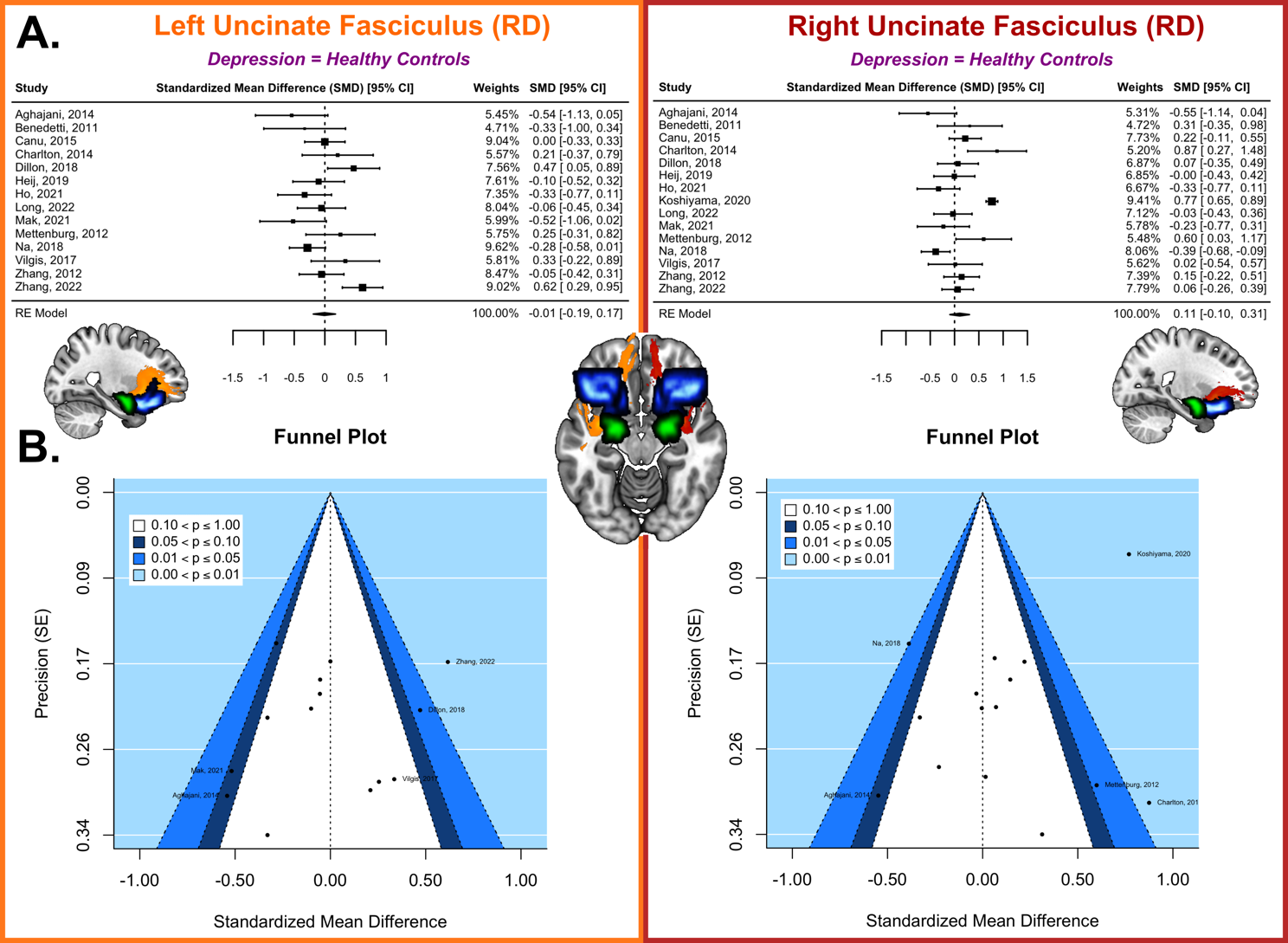
^

**A.** Forest plots, with the black diamond representing the overall effect size of each study. **B.** Funnel plots, with the dotted line representing the overall effect size. Symmetric funnel plots indicate an absence of publication bias, with a majority of studies falling in the area of statistical non-significance (p>0.1). The five most extreme points on each funnel plot are labeled with the study author and year.

**^Abbreviations: CI^**^, confidence interval;^ **^RD,^** ^radial diffusivity;^ **^RE model^**^, random-effect model;^ **^SE,^** ^standard error.^

**Figure S2. Tract-of-interest meta-analysis comparing fractional anisotropy in the uncinate fasciculus between first-degree relatives of individuals who either (1) met criteria for depression or (2) scored higher than the clinical cut-off on a dimensional depression rating scale, and healthy controls. Here, the left uncinate fasciculus (in orange) and right uncinate fasciculus (in red) are depicted. These tracts interconnect the amygdala (in green) to the orbitofrontal cortex (in blue).**

^
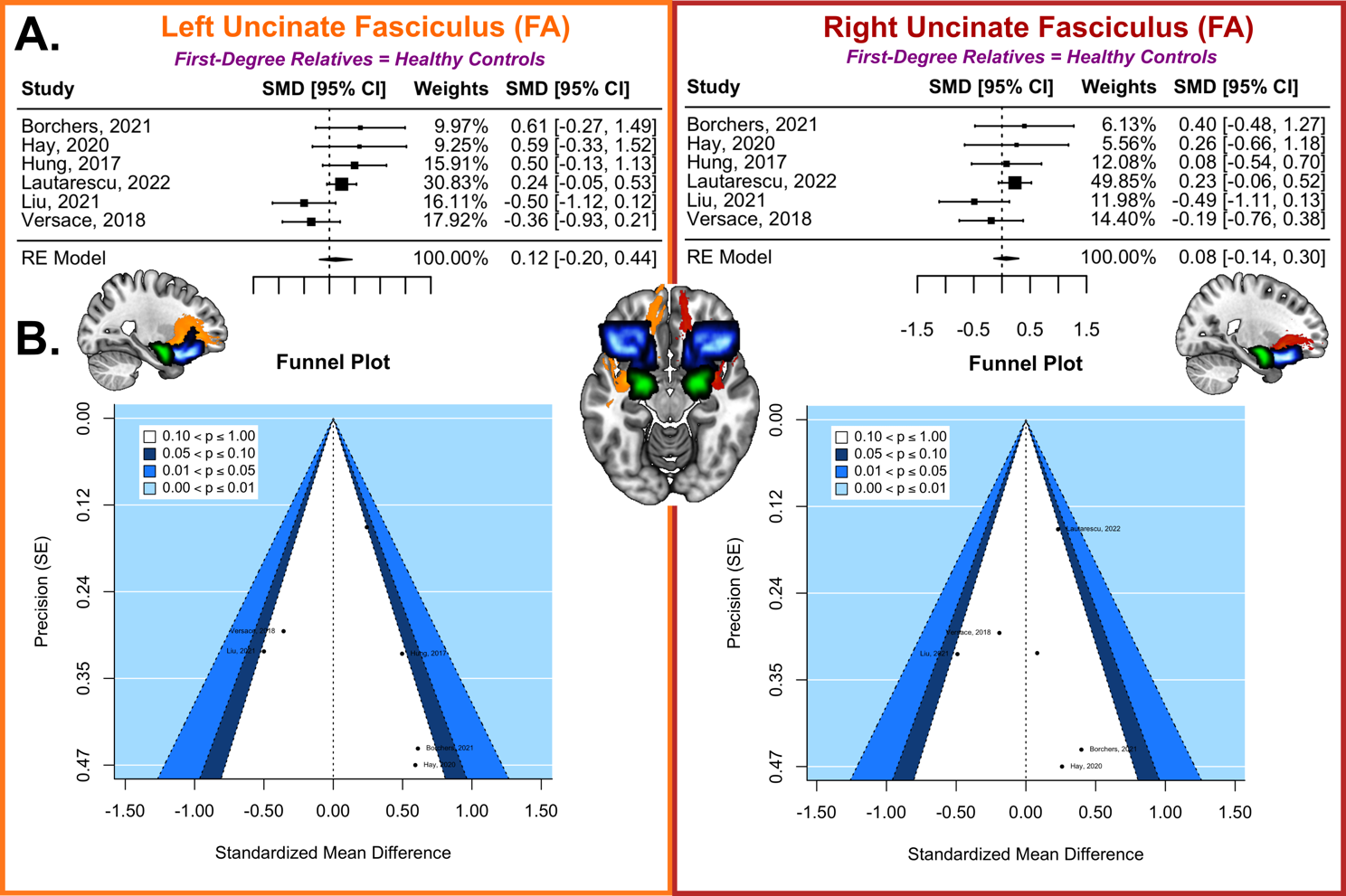
^

**A.** Forest plots, with the black diamond representing the overall effect size of each study. **B.** Funnel plots, with the dotted line representing the overall effect size. Symmetric funnel plots indicate an absence of publication bias, with all studies falling in the area of statistical non-significance (p>0.1).

**Abbreviations: CI**, confidence interval; **FA,** fractional anisotropy; **RE model**, random-effect model; **SE,** standard error.

**Figure S3. Tract-of-interest meta-analysis comparing mean diffusivity in the uncinate fasciculus between individuals with depression and healthy controls. Here, the left uncinate fasciculus (in orange) and right uncinate fasciculus (in red) are depicted. These tracts interconnect the amygdala (in green) to the orbitofrontal cortex (in blue).
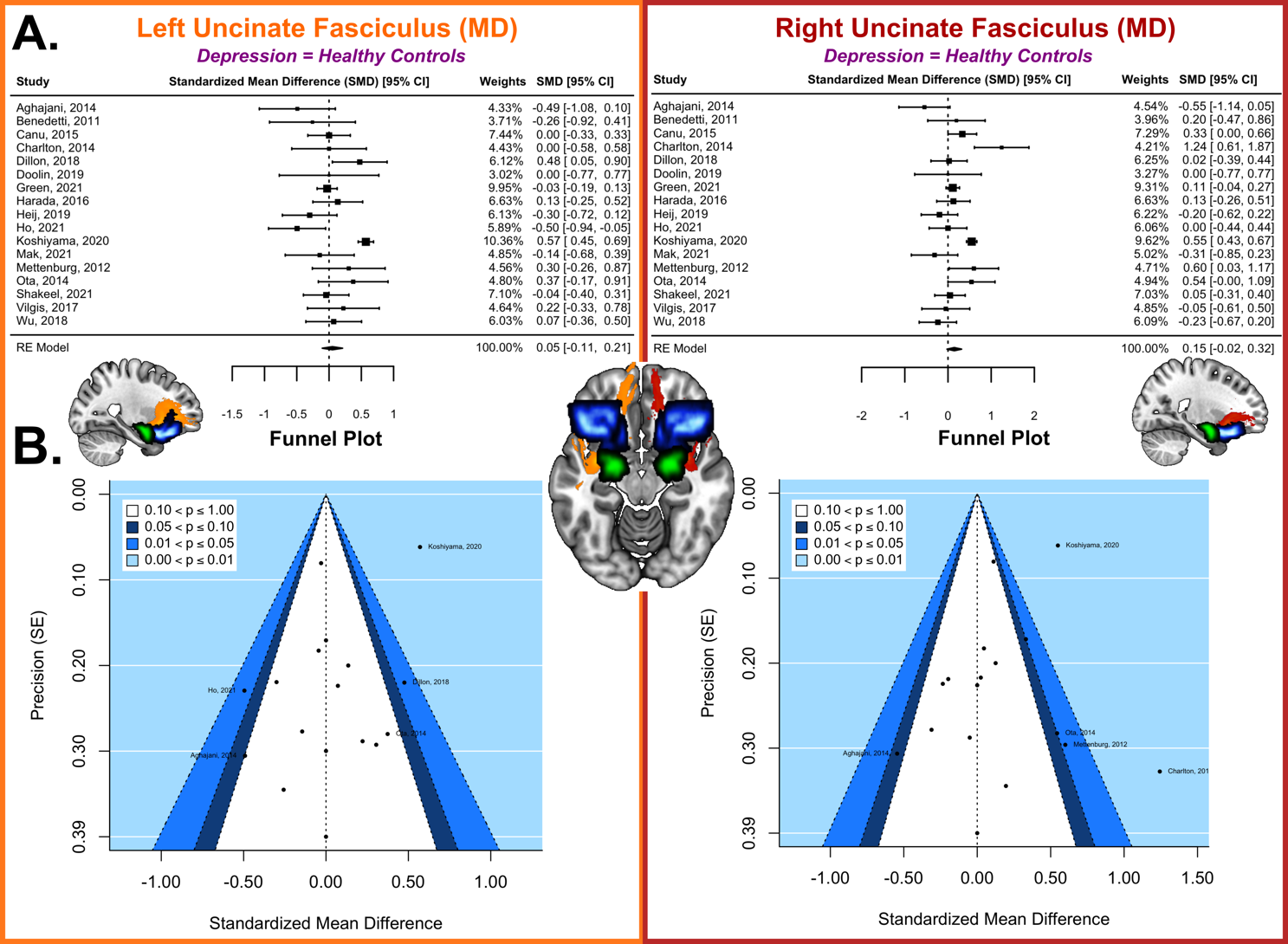
**

**A.** Forest plots, with the black diamond representing the overall effect size of each study. **B.** Funnel plots, with the dotted line representing the overall effect size. Symmetric funnel plots indicate an absence of publication bias, with all studies falling in the area of statistical non-significance (p>0.1).

**^Abbreviations: CI^**^, confidence interval;^ **^MD,^** ^mean diffusivity;^ **^RE model^**^, random-effect model;^ **^SE,^** ^standard error.^

**Figure S4. Tract-of-interest meta-analysis comparing axial diffusivity in the uncinate fasciculus between individuals with depression and healthy controls. Here, the left uncinate fasciculus (in orange) and right uncinate fasciculus (in red) are depicted. These tracts interconnect the amygdala (in green) to the orbitofrontal cortex (in blue).**

**
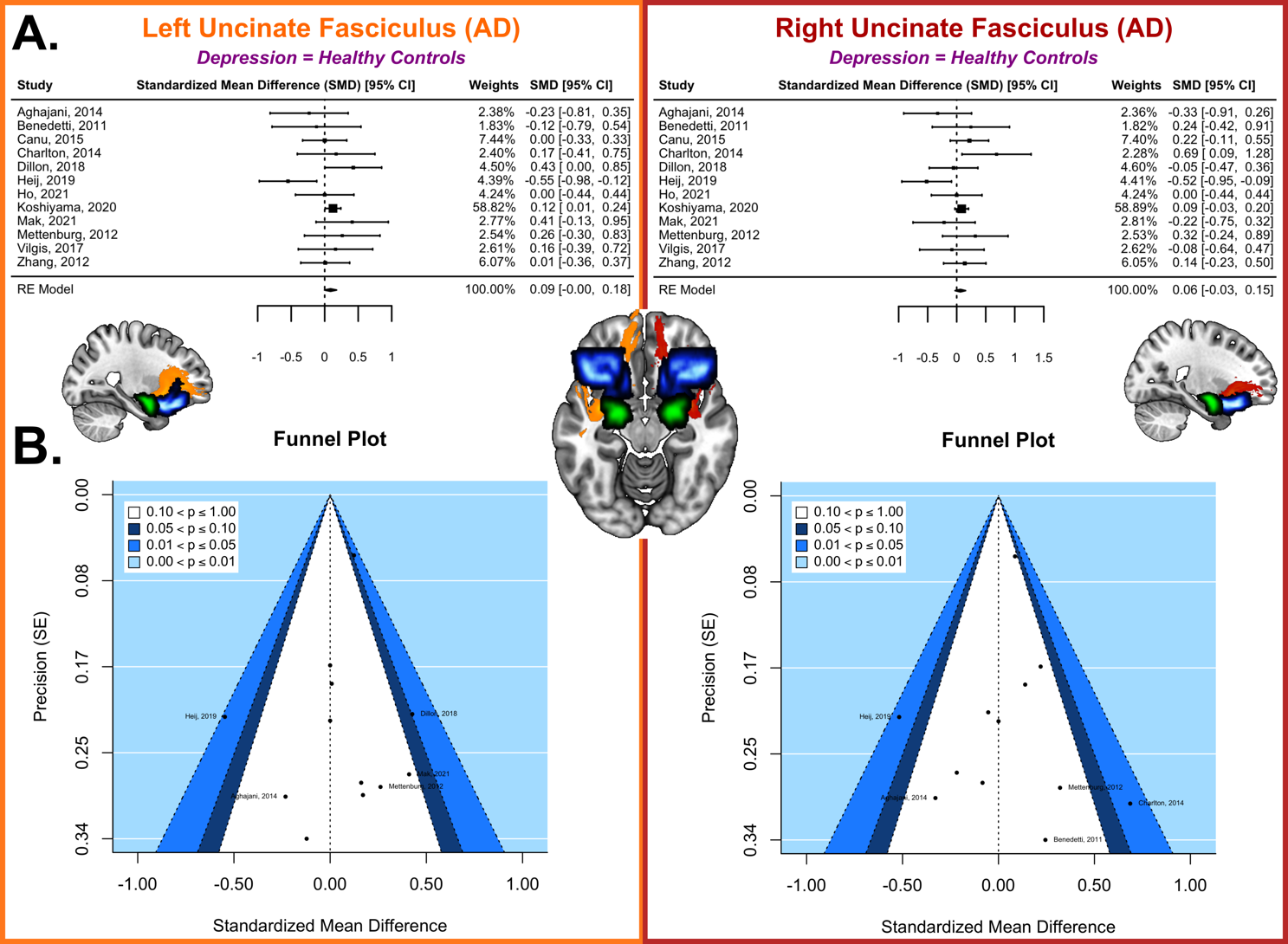
**

**A.** Forest plots, with the black diamond representing the overall effect size of each study. **B.** Funnel plots, with the dotted line representing the overall effect size. Symmetric funnel plots indicate an absence of publication bias, with all studies falling in the area of statistical non-significance (p>0.1).

**^Abbreviations: AD,^** ^axial diffusivity;^ **^CI^**^, confidence interval;^ **^RE model^**^, random-effect model;^ **^SE,^** ^standard error.^

**References**

1. Shakeel, M. K., Hassel, S., Davis, A. D., Metzak, P. D., MacQueen, G. M., Arnott, S. R., ... & Lebel, C. (2021). White matter microstructure in youth at risk for serious mental illness: A comparative analysis. P*sychiatry Research: Neuroimaging*, *312*, 111289.

2. Borchers, L. R., Dennis, E. L., King, L. S., Humphreys, K. L., & Gotlib, I. H. (2021). Prenatal and postnatal depressive symptoms, infant white matter, and toddler behavioral problems. *Journal of Affective Disorders, 282*, 465-471.

3. Hay, R. E., Reynolds, J. E., Grohs, M. N., Paniukov, D., Giesbrecht, G. F., Letourneau, N., ... & Lebel, C. (2020). Amygdala-prefrontal structural connectivity mediates the relationship between prenatal depression and behavior in preschool boys. *Journal of Neuroscience, 40*(36), 6969-6977.

4. Hung, Y., Saygin, Z. M., Biederman, J., Hirshfeld-Becker, D., Uchida, M., Doehrmann, O., ... & Gabrieli, J. D. (2017). Impaired frontal-limbic white matter maturation in children at risk for major depression. *Cerebral Cortex*, *27*(9), 4478-4491.

5. Lautarescu, A., Bonthrone, A. F., Pietsch, M., Batalle, D., Cordero-Grande, L., Tournier, J., ... & Counsell, S. J. (2022). Maternal depressive symptoms, neonatal white matter, and toddler social-emotional development. *Translational psychiatry*, *12*(1), 1-12.

6. Liu, Z., Kang, L., Zhang, A., Yang, C., Liu, M., Wang, J., ... & Sun, N. (2021). Injuries in Left Corticospinal Tracts, Forceps Major, and Left Superior Longitudinal Fasciculus (Temporal) as the Quality Indicators for Major Depressive Disorder. *Neural Plasticity*, *2021*.

7. Versace, A., Ladouceur, C. D., Graur, S., Acuff, H. E., Bonar, L. K., Monk, K., ... & Phillips, M. L. (2018). Diffusion imaging markers of bipolar versus general psychopathology risk in youth at-risk. *Neuropsychopharmacology*, *43*(11), 2212-2220.
